# 

**Published:** 1887-11-19

**Authors:** 


### CONTENTS.

**The German Crown Prince** - - - - - - 125

**Words of Consolation.**—Chapter LIX. The Sufferings of Christ - - - - - - 126

**The Doctor's Pulpit.**—Woman at Home - - - - 127

**A Woman on "Woman at Home"** - - - - - 128

**Modern Drug Laboratories.** By OUR SPECIAL COMMISSIONER - - - - - - - - 129

**Recent Drugs and Appliances** - - - - - 130

**Clinical and Therapeutic Notes.** By C R. ILLINGWORTH M.D., M.R.C.S. - - - - - - - 131

**Notes and News.**—The Spectroscope in Poisoning Cases.—Oswestry Ho p t l Dispute.—Bad Bedding and Fever.—Innoculation Experiments.—Blackburn and East Lancashire Infirmary and its Musical Friends.—Blair Convalescent Hospital.—The "Engineering and Building Record."—The Victoria Hospital for Children and Advertisements.—Metropolitan Hospital Sunday Fund.—Vacancies.—The City of Dublin Hospital and its Medical Staff—a Protest.—St. George's Hospital and its Critics.—Londonderry City and County Infirmary and its Fever Hospital, etc., etc. - - 132

**Annotations.**—Precocity and Health.—Colour-blindness in the Mercantile Marine.—More Home Claims.—A New Fever Hospital - - - - - - - - 135

**The National Pension Fund.**—Opinions of the Press - 136

**The Editor's Letter Box.**—Van Houten's Cocoa v. Whole Meal Bread.—Dr. Jaeger's Clothing - - - - 136

**East and West.** By LESLIE KEITH. Chapter VIII. An Old Friend with a New Face - - - - - - - 137

**The Hospitals Association.**—A Discussion on Hospital Construction - - - - - - - - 139

**Amusements with Profit, for all Ages.**—The Children's Corner, etc., etc. - - - - - - - 140

